# Is Graves’ disease a primary immunodeficiency? New immunological perspectives on an endocrine disease

**DOI:** 10.1186/s12916-017-0939-9

**Published:** 2017-09-25

**Authors:** Tristan Struja, Alexander Kutz, Stefan Fischli, Christian Meier, Beat Mueller, Mike Recher, Philipp Schuetz

**Affiliations:** 1 Medical University Department, Clinic for Endocrinology, Diabetes & Metabolism, Kantonsspital Aarau, Aarau, Switzerland; 2 Medical Clinic, Department for Endocrinology, Diabetes & Metabolism, Kantonsspital Luzern, Luzern, Switzerland; 3 Medical Faculty of the University of Basel, Basel, Switzerland; 4 Division of Endocrinology, Diabetes & Metabolism, University Hospital and University Basel, Basel, Switzerland; 5 Medical Outpatient Clinic and Immunodeficiency Laboratory, Department of Biomedicine, University Hospital and University Basel, Basel, Switzerland

**Keywords:** Graves’ disease, Review, Etiology, Pathophysiology, Primary immunodeficiency

## Abstract

**Background:**

Uncertainty about factors influencing the susceptibility and triggers for Graves’ disease persists, along with a wide variation in the response to anti-thyroid drugs, currently at approximately 50% of non-responders. The aim of this narrative review is to summarize immunological concepts, with a combined endocrine and immunological perspective, to highlight potential new areas of research.

**Main text:**

Relevant studies were identified through a systematic literature search using the PubMed and EMBASE databases in March 2016. No cut-offs regarding dates were imposed. We used the terms “Graves’ Disease” or “Basedow” or “thyrotoxicosis” together with the terms “etiology”, “pathophysiology”, “immunodeficiency”, “causality”, and “autoimmunity”. The terms “orbitopathy”, “ophthalmopathy”, and “amiodarone” were excluded. Articles in English, French, German, Croatian, Spanish, and Italian were eligible for inclusion.

**Conclusions:**

While concepts such as the impact of iodine, smoking, human leucocyte antigen, infections, and ethnicity are established, new ideas have emerged. Pertaining evidence suggests the involvement of autoimmunity and immunodeficiency in the pathophysiology of Graves’ disease. Recent studies point to specific immunological mechanisms triggering the onset of disease, which may also serve as targets for more specific therapies.

## Background

Primary immunodeficiency diseases (PIDs) are genetically determined diseases impacting the normal function of the immune system due to mutations in immune system genes. More than 250 PID entities have been defined to date, with the number rapidly evolving. Many patients with a PID show their first clinical manifestation in adulthood and, in some patients, the PID is never diagnosed since it is not searched for. PIDs may clinically manifest in both childhood or adulthood and patients may present with a susceptibility to infection. However, a significant number of PID patients present with autoimmune disease (AID) alone. The association of PIDs with AIDs is mechanistically complex and multifactorial (Table 1) [1]. The clinical penetrance of PID-associated gene mutations is far below 100% and novel mutations may occur during spermatogenesis or during oogenesis, implying that lack of a familial history does not argue against PIDs in a patient with autoimmunity.

**Table 1:** Interplay of autoimmunity and immunodeficiency regarding Graves’ disease

Primary immunodeficiency	Phenotype	Involved cell types	Protein/Synapse	Gene	Mechanism	Association with Graves’ disease	Reference
Selective IgA deficiency	Celiac disease, type 1 diabetes mellitus, impaired mucosal defense, most common PID	B cells Granulocytes	Ligation with its receptor (FcαRI) leads to ADCC, granulocyte degranulation, phagocytosis and neutrophil oxidative burst	Most commonly HLA haplotype 8.1	Ligation with its receptor (FcαRI) leads to ADCC, granulocyte degranulation, phagocytosis and neutrophil oxidative burst	++	[95, 96]
Severe combined immunodeficiency	Recurrent infections, vitiligo, atopic dermatitis, ITP, AH, AITD	T cells	Low expression of T cell receptor	CD3γ gene	Impaired negative selection in thymus	+	[59]
Autoimmune lymphoproliferative syndrome	Autoimmunity and polyclonal lymphocyte accumulation with lymphadenopathy and splenomegaly	T cells	Intra-thymic apoptosis via FASL to its receptor FAS (CD95) and subsequent activation of caspases 8 and 3 with impaired T cell apoptosis	Heterogeneous mutations of FAS signaling pathway	(1) Molecular mimicry or (2) Slowing down of apoptosis and/or exposure of apoptosis related autoantigens	+	[62–64]
IPEX syndrome	Immune dysregulation (eczema), polyendocrinopathy (T1DM), enteropathy	Treg cells Thymus	Loss of essential transcription factor	FOXP3 on X chromosome	Greatly reduced Treg cell number	++	[65]
IPEX-like	IPEX-like phenotype	Treg cells	IL-2-receptor-α chain (CD25)	CD25 deficiency due to autosomal recessive mutations	Normal Treg in numbers but deficient stimulation by defective IL-10 expression	+/–	[67–70]
Common variable immunodeficiency	Various autoimmune diseases (ITP, AIHA, psoriasis, AITD, arthritis) and antibody deficiencies,organ infiltration (bone marrow, kidney, brain, liver, spleen) by granulomatous-lymphocytic infiltration	APCs Treg cells B cells Naïve T cells	CTLA-4 binds to CD80/CD86 (B7) on APCs, leading to lower levels of co-stimulatory B7, failure to activate CD28 (the ligand for B7) on T cells	CTLA-4 germline mutations with incomplete penetrance	Treg cells increased, but dysfunctional (decreased CTLA-4 ligand binding)	+	[71, 75]
Systemic lupus erythematosus -like	Recurrent infections; cutaneous, discoid lupus most common presentation, malar rash, oral ulcers, recurrent fever and vasculitis	Macrophages Apoptotic B cells	MFG-E8 (mice) and complement factor C1q	Homozygous nonsense and missense mutations on chromosome 1p (C1q)	Impaired debris removal, autoantibodies against C1q correlates with thyroid function in AITD	+	[76–79]
Common variable immunodeficiency	See above	APCs Treg cells B cells naïve T cells	BAFF BAFF-R P21R variant	BAFF (SNPs rs1041569 & rs2893321)P21R (TNF-RSF13C allele)	Higher levels stimulate B cell survival, increase of TR antibody levels	++	[102–104, 106, 107]
Hyper-IgM syndrome	Elevated serum IgM, but deficiency in IgG/A/E, recurrent respiratory and gastrointestinal infections with pyogenic bacteria and opportunistic organisms (e.g., *P. jirovecii*)	APCs T cells B cells Thyrocytes	CD40	Autosomal recessive CD40 gene mutations	Upregulation of CD40 on thyrocytes, increased co-stimulatory effects and immunoglobulin class switching	+	[5, 14, 54, 108]
Common variable immunodeficiency	See above	APCs Treg cells B cells Naïve T cells	Miscellaneous	Decreased methylation of various genes	Higher ICAM-1, decreased B cell class switching	+/–	[109, 120, 121]
Skewed X-chromosome inactivation	Wiskott–Aldrich syndrome: PID with eczema, thrombocytopenia, and diarrhea	Thymus T cells B cells	Miscellaneous	Genes for Wiskott–Aldrich syndrome protein, CD40L or the IL-2 receptor-$\gamma$ chain	Reduced thymic expression of X chromosome-dependent self-antigens primes inadequate T cell apoptosis	++	[118, 119]
Trisomy 21	Down syndrome: increased susceptibility to leukemia, but reduced incidence of solid tumors	Thymus T cells	IFN-γ	AIRE and FOXP3 on X chromosome	Increased production of IFN-γ with augmented Th1 responses Reduced activity of Treg cells	++	[51, 53, 66]

*ADCC* antibody dependent cellular cytotoxicity, *AIHA* autoimmune hemolytic anemia, *AITD* autoimmune thyroid disease, *AH* autoimmune hepatitis, *APC* antigen presenting cell, *BAFF* B-lymphocyte activating factor, *CD* cluster of differentiation, *CTLA* cytotoxic T-lymphocyte-associated protein, FasL type-II transmembrane protein of TNF family, *FOXP3* forkhead box P3, *HLA* human leukocyte antigen, *IL* interleukin, *IFN* interferon, *IPEX* immunodysregulation polyendocrinopathy enteropathy X-linked, *ITP* immune thrombocytopenic purpura, *MFG-E8* Milk fat globule epidermal growth factor 8, *PID* primary immunodeficiency, *T1DM* type 1 diabetes mellitus, *TR* thyrotropin related

## Main text

### Literature search and inclusion criteria

Relevant studies were identified through a systematic literature search using the PubMed and EMBASE databases in March 2016. No cut-offs regarding dates were imposed. We used the terms “Graves’ Disease” or “Basedow” or “thyrotoxicosis” together with the terms “etiology”, “pathophysiology”, “immunodeficiency”, “causality”, and “autoimmunity”. The terms “orbitopathy”, “ophthalmopathy”, and “amiodarone” were excluded. Articles in English, French, German, Croatian, Spanish, and Italian were eligible for inclusion.

### Immunologic view on exogenous factors associated with Graves’ disease (GD)

In iodine-sufficient areas, GD is the most common cause of primary hyperthyroidism in the younger population [2]. It is an autoimmune disorder characterized by the presence of thyrotropin-related antibodies (TRAb) that stimulate thyroidal cells, resulting in an overproduction of thyroid hormones [3].

The global prevalence of GD has been reported to range from 0.5% to 2% of the population, with an incidence of 1 in 4000 persons per year [4, 5]. Similar to other AIDs, GD is predominantly found in females – with a female-to-male ratio of four to six – and occurs predominantly in the third and fourth decade of life [5]. Age is an important factor determining the response to treatment, as indicated by a recent report showing that the overall remission rate after anti-thyroid drug (ATD) treatment in a cohort of 268 children was 15% [6], markedly lower than the 40–60% usually seen in adults [4]. As GD tends to relapse more often in the young, it can be speculated that these individuals harbor a strong genetic susceptibility. This notion is supported by the finding that monozygotic twins share concordance for GD in roughly 75% of cases [7–10]. Geographic variations in the incidence of GD may be due to genetic variations and/or exposure to local factors, but immigrants from lower risk regions acquire a similar risk over time as people in the new area of residence [11]. Analyses of National Health and Nutrition Examination Survey data from the United States have also demonstrated racial variations in prevalence; indeed, African Americans were more likely to develop thyrotoxicosis (odds ratio (OR) 2.9, 95% confidence interval 1.5–5.7) compared to Caucasians, whereas there was no difference in prevalence between Hispanics and Caucasians [12].

**Fig. 1:**
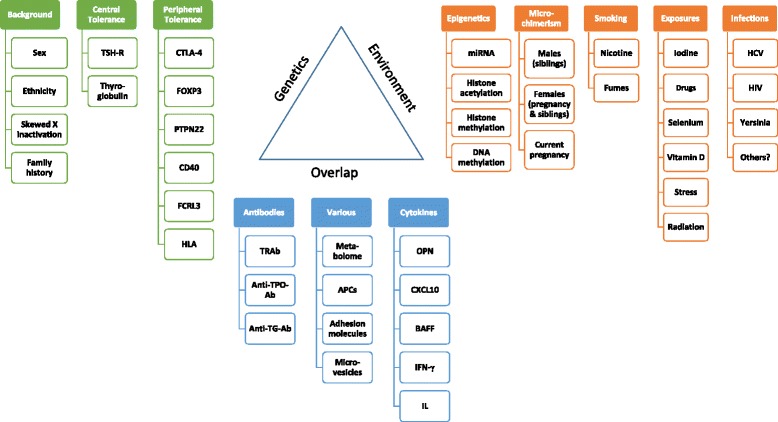
Interplay of factors influencing the pathophysiology of Graves’ disease. *Ab* antibodies, *APC* antigen presenting cell, *BAFF* B-lymphocyte activating factor, *CD* cluster of differentiation, *CTLA* cytotoxic T-lymphocyte-associated protein, *CXCL* C-X-C motif chemokine, *FCRL* Fc receptor-like protein 3, *FOXP3* forkhead box P3, *HCV* hepatitis C virus, *HIV* human immunodeficiency virus, *HLA* human leukocyte antigen, *IL* interleukin, *IFN* interferon, *OPN* osteopontin, *PTPN* protein tyrosine phosphatase, *TG* thyroglobulin, *TPO* thyroperoxidase, *TRAb* thyrotropin related antibodies, *TSH-R* thyroid-stimulating hormone receptor

**Fig. 2:**
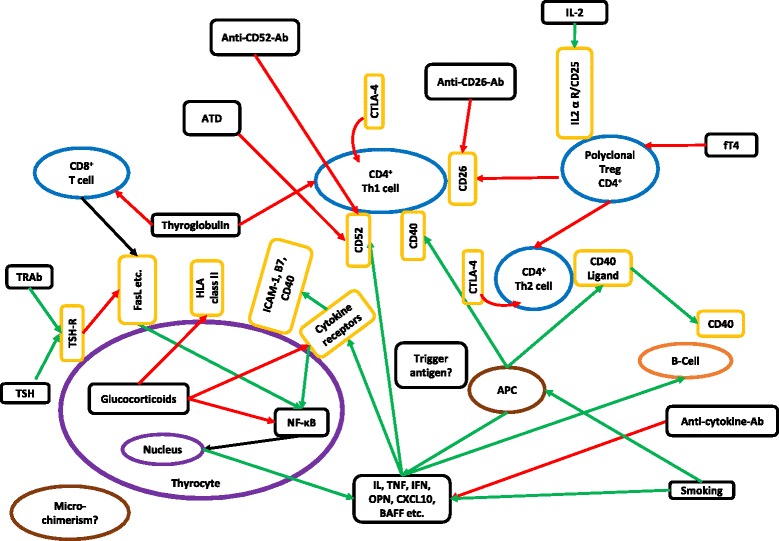
Interplay of etiological factors associated with Graves’ disease at the molecular level. Green arrows, stimulatory effects; red arrows, inhibitory effects; purple, thyrocytes; yellow, membrane receptors; blue, T cells; light brown, B cells; dark brown, miscellaneous. *Ab* antibodies, *APC* antigen presenting cell, *ATD* anti-thyroid drug, *BAFF* B-lymphocyte activating factor, *CD* cluster of differentiation, *CTLA* cytotoxic T-lymphocyte-associated protein, *CXCL* C-X-C motif chemokine, *FasL* type-II transmembrane protein of TNF family, *fT4* free thyroxine, *HLA* human leukocyte antigen, *ICAM-1* and *B7* adhesion molecules, *IL* interleukin, *IFN* interferon, *NF-κB* nuclear factor kappa-light-chain-enhancer of activated B cells, *OPN* osteopontin, *TNF* tumor necrosis factor, *TRAb* thyrotropin related antibodies, *TSH-R* thyroid-stimulating hormone receptor

The overall effect size for genetic markers in most studies is rather weak, with OR ranging from 2 to 4 for genes in the human leucocyte antigen (HLA) complex and from 1.1 to 2.6 for other genes [13–15], thus providing evidence of the involvement of other factors [14]. Various factors have been suggested to be causative (Figs. 1 and 2) of GD, and will be discussed in more detail in this review. The great disadvantage in all these genome-wide association studies (GWAS) is the fact that GD is likely a heterogeneous disease and might represent a common final path of various and different gene mutations. Therefore, the pooling of patients with various genetic etiologies of GD will dilute the impact of specific gene mutations.

#### Environmental and dietary factors

Many environmental influences that may alter the immune system and have an impact on the pathogenesis of GD have so far been identified (Fig. 1).

An increased incidence of GD in the postpartum period has long been known [16, 17]. During pregnancy, increased immune-regulation occurs to avoid immune attack towards the haplo-identical fetal tissue through a shift from a type 1 helper T cell response (Th1, responsible for immune rejection of non-matched organ transplants) to a type 2 helper T cell (Th2) response that favors antibody production. Thus, theoretically, this could favor autoantibody and therefore TRAb production [17]. Further, there is increased activity of regulatory T cells (Treg) during pregnancy, impairing auto-immune responses via several different pathways [18, 19]. This immuno-suppressive milieu is abrogated after birth, which might explain the onset of autoimmunity in the postpartum period.

Alemtuzumab, a humanized anti-CD52 antibody originally designed for the treatment of chronic lymphocytic leukemia, is associated with a higher risk for onset of GD [20]. Alemtuzumab depletes both T and B lymphocytes and thus creates a drastic immunodeficient state. This clinical association demonstrates, as a proof of concept, that immunodeficiency and autoimmunity are two sides of the same coin. Alemtuzumab-induced B cell depletion leads to a reactive increase in the B-lymphocyte activating factor (BAFF) in serum. BAFF promotes survival of B cells in general but of autoimmune B cells in particular and thus favors autoantibody production [21–23]. In addition, the BAFF-driven B cell reconstitution following alemtuzumab treatment leads to a transient phase where most of the peripheral blood B cells have an immature, transitional B cell phenotype. Transitional B cells carry B cell receptors with a higher reactivity against self-antigens compared to mature, naïve B cells [24].

An association of GD with smoking has been found in several studies, with a meta-analysis reporting an OR of 3.30 (95% confidence interval 2.09–5.22) [25] and being more pronounced in women [26]. Smoke may damage cells directly, leading to increased accessibility of thyroid gland-derived auto-antigens and may also alter levels of numerous cytokines and soluble cytokine receptors [11]. Another possibility might be the induction of epigenetic changes through smoking [27]. Nicotine, per se, has immunologic functions through the binding to cholinergic receptors on monocytes. Nicotine downregulates the release of innate immune system stimulating high-mobility group box 1 protein by monocytes [28].

Selenium supplementation has been shown to have beneficial effects on mild Graves’ orbitopathy [29]. Yet, whether there are also protective effects on GD relapse rates remains unclear, but an ongoing randomized controlled trial might shed more light on this topic [30]. Selenium deficiency is thought to favor autoimmunity by tipping the Th cell balance towards a Th1 predominance, increased pro-inflammatory cytokines, and reducing the number of Treg cells [31].

Exposure to radiation, such as neck irradiation or nuclear fallout, can prime AIDs through destruction of thyrocytes with consequent release of otherwise immunologically ignored antigens [32]. Radiation of the thymus may also impair thymus function and thus the generation of Treg cells. In a mouse model, the latter mechanism seemed to be triggering radiation-induced autoimmunity rather than radiation-induced release of organ-derived antigens [33]. Nevertheless, results are contradictory as some studies report an increased incidence of GD following irradiation whereas others do not [34–36].

#### Infections

GD has been reported to occur at a higher frequency after a mean of 17 months following initiation of highly active antiretroviral therapy (HAART) in HIV patients [37]. This coincides with HAART-induced immune reconstitution, as a sequela of massive production of naïve T cells from the thymus. IL-7 may be the T-cell counterpart of the aforementioned BAFF, as it is highly increased in states of T cell lymphopenia [38] and is involved in survival/proliferation of autoreactive T cells [39]. Recently, a therapeutic trial of three doses of recombinant IL-7 applied to increase peripheral T cell numbers in patients with T cell lymphopenia led to induction of systemic lupus erythematosus (SLE) in one out of nine patients [40]. At the same time, immune reconstitution is associated with a lower number of Treg cells, which may further increase the risk of autoimmunity [41].

In the late 1970s, an increased rate of infection with *Yersinia enterocolitica* in patients with GD was discovered [42–44], but the cross-reacting proteins had not been identified until recently [45]. In 2013, Hargreaves et al. [45] showed that TRAb acquire activity only after early precursor B cells undergo somatic hyper-mutation and the mutated antibodies begin to recognize the outer membrane porins A, C, and F of *Y. enterocolitica*. The authors speculate that memory B cells acquire cross-reactivity with the ability to recognize both the initial pathogen and the thyroid-stimulating hormone (TSH) receptor (TSH-R).

Besides its association with cryoglobulinemic vasculitis and Sjögren’s syndrome, infection with hepatitis C (HepC) virus predisposes to GD [46]. Interestingly, autoimmune thyroid diseases, and GD in particular, are more frequent in patients with HepC as compared to HepB patients following treatment with interferon (IFN) [47]. One reason for this might be that the HepC virus and thyroid proteins share homologies [48]. The link of type I IFNs to AID is most strikingly demonstrated in a subgroup of monogenic PIDs that share an overexpression of IFN-regulated genes, the so-called interferonopathies [49]. Many of those clinically share chilblain lupus-like skin lesions and calcifications in the central nervous system. A recent analysis of autoimmunity in these patients also described autoimmune thyroiditis in a boy with a genetically proven monogenic interferonopathy [50].

In this respect, it is very interesting that patients with trisomy 21 have a higher incidence of GD [51, 52] and show many features of an interferonopathy. This may be partly linked to the fact that the IFN type I receptor is located on chromosome 21. This increased gene load of IFN-Rs may lead to a constant activation of the IFN pathway [53].

### Immunologic view on genetic factors associated with GD

#### Association with HLA

HLAs on chromosome 6, which are highly polymorphic between individuals, have a pivotal role within the immune system. HLA-A to -C (MHC class I) molecules present antigens to CD8+ T cells and are expressed on almost all tissues. HLA-D (MHC class II) molecules present their antigens to CD4+ T cells and are primarily expressed on antigen presenting cells (APCs) such as dendritic cells or B cells [54]. As already stated, they confer the highest susceptibility for GD of all identified genes based on GWAS analyses [14]. Their sequence diversity largely results from mutations altering the antigen-presenting pocket of the MHC protein. Thus, one hypothesis holds that genetic alterations in regions coding for the structure of the pocket lead to the MHC-presentation of autoantigens to CD4^+^ T cells. Moreover, interactions between HLA receptors and autoantigens may further multiply the possibilities. This is supported by the fact that the thyroglobulin (*TG*) gene itself contains GD-susceptible polymorphisms [55, 56]; only certain TG peptides can bind strongly to a corresponding disease-susceptible HLA-DR3 pocket, whereas others do not. Moreover, this results in reduced thymic-negative selection of autoreactive T cells [57, 58].

#### Mutations associated with impaired central T cell tolerance

T cells whose T cell receptor (TCR) recognizes autoantigens are deleted by apoptosis in the thymus in a process called negative selection. This process requires correct expression of the TCR and normal T cell signaling. CD3 is expressed on all T lymphocytes and critically regulates TCR signaling, thereby regulating the response to antigen encounters as well as positive and negative selection in the thymus. Mutations in the *CD3γ* gene on peripheral T cells lead to low expression of TCR and thus to impaired negative selection of autoimmune T cells in the thymus. Nine patients with familial autoimmune thyroiditis with deficiencies in *CD3γ* have been identified to date [59]. These patients also have susceptibility to infections due to diminished T cell stimulation by antigens [59]. The protein tyrosine phosphatase-22 gene (*PTPN22*) encodes lymphoid tyrosine phosphatase, which suppresses TCR signaling on central and peripheral T cells in vitro via the C-src tyrosine kinase and halts IL-2 production in mice [54]. Consequently, expansion of T cells is inhibited. Loss-of-function mutations in *PTPN22* increases GD susceptibility, with an OR of 1.5 to 1.9 [54]. However, the exact mechanism by which this occurs has not been identified to date, and associations between these loss-of-function mutations in *PTPN22* and the incidence of GD vary considerably between ethnicities [54, 60].

The autoimmune regulator (AIRE) is a transcription factor that ensures thymic expression of normally extrathymic antigens such as insulin. Human AIRE deficiency leads to organ-specific autoimmunity, and is believed to be a consequence of dysfunctional central immunologic tolerance. AIRE deficiency is often associated with autoimmune adrenalitis; however, GD is not associated with AIRE polymorphisms [61].

Thymic negative selection occurs by activation-induced apoptosis of T cells that recognize autoantigens with their TCR. This process is executed by the binding of FASL to its receptor FAS (CD95) and subsequent activation of caspase 8 and 3, which then leads to T cell apoptosis. Mutations in FAS and FASL lead to autoimmune lymphoproliferative syndrome, a primary immunodeficiency associated with autoimmune cytopenia but also additional autoimmune features. Interestingly, it has been shown that 24/28 patients with GD have impaired in vitro T cell apoptosis following FAS ligation [62].

In GD, it has been shown that intra-thyroid T cells release apoptosis-inhibiting factors such as caspase 8 inhibitor protein and antiapoptotic BCL-proteins [63]. This might explain why thyrocytes from GD patients survive the autoimmune T cell response, whereas those from patients with Hashimoto’s thyroiditis (HT) do not [64].

#### Mutations associated with impaired peripheral T cell tolerance

Autoreactive T cells can be found in the peripheral blood of healthy persons and are kept in check by Treg cells that inhibit, through various mechanisms, autoreactive T cell activation. These Treg cells express the transcription factor forkhead box P3 (FOXP3), which is essential for the generation and maintenance of Treg cells.

Mutations in FOXP3 (located on the X chromosome) lead to immunodysregulation polyendocrinopathy enteropathy X-linked (IPEX) syndrome, a systemic AID immunologically characterized by the loss of Treg cells and clinically manifesting as eczema, enteropathy, and autoimmune endocrinopathy, which may include both hypo- and hyperthyroidism [65].

As outlined earlier, patients with trisomy 21 do not only have an increased incidence of GD, but also an earlier disease onset and more associated AIDs as compared to the general population [52]. However, this cannot be solely attributed to the constitutional activation of the IFN pathway – although these patients exhibit an increased number of Treg cells with normal proliferative capabilities in peripheral blood, their cells have a markedly reduced inhibitory activity [66].

Human Treg cells are the only T cells that express high levels of the IL-2 receptor α chain (CD25) in the naïve (non-activated) state; IL-2 is essential in maintaining the normal life cycle of Treg cells. Human CD25 deficiency causes an IPEX-like phenotype [67]. Further, Treg cells have been shown to be lower in numbers and to be dysfunctional in patients with GD [68]. A polymorphism in the *CD25* gene was found to be associated with GD in untreated Russian patients [69], whereas these results could not be replicated in Han Chinese [70].

Treg cells acquire regulatory/suppressive function through the high expression of CTLA-4. CTLA-4 binds to CD80/CD86 (B7) on APCs and may trans-endocytose it [71]. Therefore, APCs express lower levels of the co-stimulatory B7 proteins and fail to activate CD28 (the ligand for B7) on T cells. Thus, the lower the expression of CTLA-4 on Treg cells, the lower the number of activated naïve T cells. This principle has already been introduced into clinical practice in the form of ipilimumab, a CTLA-4 blocking monoclonal antibody that boosts the activation of melanoma-specific T cells [72]. However, therapeutic use of ipilimumab has been shown to induce autoimmune hyperthyroidism [73]. The programmed cell death protein 1 has similar functions as CTLA-4 and its blockade also causes GD [74]. CTLA-4 expression may be lowered due to heterozygous germline mutations (CTLA-4 haploinsufficiency). Thus, these patients do not only develop various autoimmune and lymphoproliferative diseases, but often have antibody deficiency. Therefore, although the numbers of peripheral Treg cells may even be increased in these patients, they are dysfunctional since they display decreased CTLA-4 ligand binding and impaired trans-endocytosis of CD80 and CD86 from APCs [75].

#### Mutations in the cell debris removal machinery

It is vital that debris from apoptosis, necrosis, and circulating immune complexes are properly disposed of throughout life. This system is maintained by proteins such as MFG-E8 and complement factor C1q [76].

A deficiency in the removal machinery of apoptotic cells leads to systemic lupus-like autoimmunity. This is exemplarily shown in lupus-like disease in humans with C1q deficiency [77] and in mice deficient for MFG-E8 [78]. Interestingly, autoantibodies against C1q correlate with thyroid function in autoimmune thyroid disease [79].

Microvesicles are released into the circulation from multiple cell types during apoptosis and reflect metabolic stress. A very recent case–control study from Sweden investigated the number of microvesicles (i.e., diameter < 1 μm) in the blood of patients with GD, which was seen to be increased in the blood of GD patients compared with healthy controls and to remain elevated after medical therapy, albeit at a lower level [80]. This concept might as well offer a new biomarker to guide future treatment decisions in patients with GD.

#### Increased IFN levels

Although it has been known since 1962 that TRAb have a causal role in hyperthyroidism, it took almost another 30 years until the precise nature of their involvement was further elucidated [81]. Interestingly, anti-TG and anti-thyroperoxidase antibodies can precede autoimmune thyroid pathologies by years, while TRAb appear in serum only a few months prior to a diagnosis of GD [3]. This underscores the fact that their involvement occurs only at the very end of the autoimmune reaction culminating in the onset of GD. TRAb can have stimulating (TSAb), blocking (TBAb), or neutral (TBII) abilities. Nevertheless, roughly 5% of patients are negative for these antibodies (even those positive for anti-thyroperoxidase antibodies) [82]. Most researchers believe that these patients have circulating antibodies that are not detected by currently available assays or that production is confined to the thyroid gland and adjacent lymph nodes [83, 84]. Furthermore, it has been shown that TRAb-negative patients have less severe hyperthyroidism and fewer of them subsequently develop Graves’ orbitopathy [83].

Based on these facts, GD has long been viewed solely as a Th2-induced B cell illness. However, evidence outlined below suggests that the initial phase is governed predominantly by Th1 cells infiltrating the thyroid. IFN-γ and TNF-α produced by APCs and B cells stimulate the secretion of C-X-C motif chemokine 10 (CXCL10) by thyrocytes in vitro [85]. It is believed that CXCL10 may then attract Th cells by binding to its cognate receptor (CXCR3), and that this effect is more prominent in patients with higher CXCL10 serum levels [86]. These T cells cross-react with B cells for the production of TSAb, constituting the IgG1 subclass [82]. This concept is supported by the fact that induction of GD by TSH-R-encoding adenoviruses is dampened if the genes encoding IL-4 or IFN-γ are absent in corresponding knockout mice, indicating that both Th1 and Th2 cytokines are needed [87]. In addition, CXCL10 serum levels are much higher in GD patients in the active phase of the disease and decrease during treatment with ATD both in vitro and in vivo and after radioactive iodine therapy. Serum CXCL10, in particular, drops dramatically after thyroidectomy, suggesting that thyrocytes are the main source of its production [3].

Later on in the mechanism, intrathyroidal Th2 cells inhibit Th1 responses through the secretion of IL-10, IL-5, and IL-4 [88]. These cytokines might prevent autoimmune destruction of the thyroid gland by placing Th1 cells into an anergic state and inhibiting macrophage activation by preventing further IFN-γ secretion [89]. Furthermore, they might protect thyrocytes from apoptosis via the Fas/FasL (CD95/CD95L) system through upregulation of two anti-apoptotic proteins, BCL-XL and cFLIP, leading to increased production and release of the caspase 8 inhibitor protein into the serum [63, 89]. This mechanism might explain why thyrocytes from GD patients survive the autoimmune response, whereas those from patients with HT do not [64]. Additionally, the increased Th2 response leads to an increased production of TBAb, composed by all IgG subclasses [88].

Another novel cytokine is osteopontin (OPN), first discovered in 1986 in osteoblasts, but not until recently has its role in AID been further elucidated. OPN is released by various cell types, including chondrocytes, dendritic cells, T cells, and macrophages, after stimulation by pro-inflammatory events or cytokines (i.e., angiotensin II, TGF-β, TNF-α, IL-1b, nitric oxide, hyperglycemia, and hypoxia) [90]. It exerts its effects via the NF-κB pathway and consecutive production of IFN-γ and IL-12 stimulating Th1 cells. Further, OPN secreted from peripheral blood mononuclear cells (PBMCs) also induces expression of CD40L on Th cells in vitro, which in turn stimulates B cells [91, 92]. Thus far, there are only a few case–control studies analyzing the effect of OPN on the development of GD. Its production seems to be positively correlated with the CCL20 cytokine secreted by Th cells in the plasma of GD patients [93]. Additionally, OPN can induce expression of CCL21 from cultured PBMCs in a dose- and time-dependent manner [94], and its plasma levels are positively correlated with biochemical disease severity in GD [90]. Taken together, these results suggest that OPN could be used as a biomarker of GD.

#### Mutations affecting B cell function – primary antibody deficiencies

Selective IgA-deficiency is the most prevalent form of antibody deficiency worldwide. The diagnosis is made when IgA is not detectable in serum (<0.07 g/L, 10-fold lower than the lower limit of serum IgA in healthy controls), while serum IgG and IgM are normal. Selective IgA deficiency is particularly common in Caucasians, with a prevalence of roughly 1 in 500 in Europe [95]. Although many patients with selective IgA deficiency do not have symptoms, some display recurrent infections of the gastrointestinal tract and airways. Of note, considerable numbers of subjects develop AIDs such as celiac disease, type 1 diabetes mellitus, SLE, and GD [95]. Selective IgA deficiency is not due to mutations in the gene encoding for IgA, but is associated with specific HLA genotypes [96]. A recent GWAS analysis showed association of selective IgA deficiency with alterations in various genes involved in intestinal IgA production and Treg cell function [97]. The association of selective IgA deficiency with Treg cell function might partly explain the clinical association with AIDs in general and GD in particular.

A highly debatable aspect may provide a plausible connection between GD and immunoglobulin G4-related disease, which represents a newly defined AID sub-entity that can affect various tissues and whose pathogenesis is still poorly understood [98]. Diagnostic criteria are an elevated serum IgG4 and high numbers of IgG4-positive plasma cells in biopsies. Increased IgG4 levels in sera from GD patients have been reported [99]. Importantly, IgG4 levels may be falsely measured as too low due to a ‘hook’ effect in current assays [100]. On the other hand, a recent extensive report did not find the typical histopathological changes in thyroid samples from GD patients that would be needed to fulfill diagnostic criteria [101].

As mentioned above, BAFF is a B cell survival factor that may be involved in the pathogenesis of GD [102]. In a mouse model of GD, administration of a scavenging BAFF antibody led to amelioration of hyperthyroidism and reduction of TRAb levels in the blood, whereas plasma cell counts in the spleen were unchanged [103]. In humans, BAFF serum levels in GD patients are elevated compared to healthy controls [104]. Interestingly, BAFF levels decline in patients after treatment with corticosteroids [105]. Recently, the BAFF rs2893321 single nucleotide polymorphism (SNP) was described and shown to increase the risk of GD, showing an association with lower TRAb levels in men, but not in women [106]. Similarly, a polymorphism in the BAFF receptor at amino acid position 21 (P21R) has been functionally analyzed in detail [107]. This polymorphism results in a BAFF receptor expressed as a monomer instead of a trimer, and hence promotes a reduced response to BAFF signals. The P21R variant is enriched in cohorts of patients with antibody deficiency and is under-represented in patients with chronic lymphatic leukemia. Nevertheless, whether the P21R BAFF receptor variant is associated with GD has not yet been analyzed.

CD40, which is present on many APCs including macrophages and B cells, belongs to the TNF-receptor superfamily and provides co-stimulatory effects following binding to CD40L/CD154 on activated T cells, inducing immunoglobulin class switching [5]. Loss-of-function mutations in the CD40L/CD40 pathway lead to hyper-IgM syndrome [108]. These patients can present with AIDs as well as with recurrent infections. Several SNPs in CD40/CD40L increase vulnerability to GD by upregulation of CD40 on thyrocytes and/or B cells, leading to amplified secretion of pro-inflammatory cytokines [14, 54].

Many new concepts concerning immune tolerance and autoimmunity have emerged in recent years, and these have been illustrated in Fig. 2. In our view, a single ideal target that would be a therapeutic breakthrough has not been tackled so far.

### Immunologic view on epigenetic factors associated with GD

As mentioned above, concordance of GD in monozygotic twins is 75%, indicating that epigenetic modifications have an important role in GD. A significant impact of epigenetic modifications has been described in patients with PIDs. Indeed, a recent in-depth analysis of epigenetic modifications in two monozygotic twins discordant for antibody deficiency showed a gain in DNA methylation of several genes critically involved in B cell function in the twin with antibody deficiency [109].

In general, epigenetic changes (for instance, histone methylation) can either intensify or reduce gene expression, depending on the number and location of the added methyl groups. This process is executed by histone methyltransferases, which use S-adenosyl-methionine to add up to three methyl groups on lysine or arginine residues [110]. In patients with GD, one report found an interaction between a non-coding homozygous SNP in intron 1 of the TSH-receptor gene in human thymus cells (from children who underwent thoracic surgery) associated with reduced TSH-R expression and histone methylation patterns that could be influenced by the addition of IFN-α to cell cultures [111]. This mechanism could expedite the release of auto-reactive T cells from central tolerance. Especially in the case of the TSH-receptor gene, this leads to diminished apoptosis of autoreactive T cells in the thymus [15, 112, 113].

Micro-RNAs are also involved in epigenetic regulation. MicroRNAs are usually 22 nucleotides long and decrease translation of a given gene by interfering with its mRNA, either by cleaving mRNA when both sequences match fully, or by simply repressing translation if the sequences only match partially [114]. Using microRNA assays, one analysis showed that the number and function of CD4^+^ CD25^+^ FOXP3^+^ Treg cells was significantly reduced in the blood of GD patients [115]. Likewise, the retinoic acid pathway was suppressed in those Treg cells and could be restored by in vitro application of the agonist all-trans retinoic acid.

Positively charged histones that strongly bind to the negatively charged DNA and hamper DNA transcription represent another possible mechanism of epigenetic transformation. Acetylation of lysine residues at the ends of the histones decreases their positive charge and promotes DNA transformation from its condensed heterochromatin to its unfolded euchromatin structure [116]. The process is facilitated by histone acetyltransferase and histone deacetylase, with the acetyl groups being provided by acetyl coenzyme-A. We found only one study evaluating the extent of histone acetylation in GD [117], where a decreased histone 4 acetylation in PBMCs was reported. The clinical significance of this finding is yet unclear, as patients with many other AIDs present with altered acetylation patterns.

DNA methylation also serves to inactivate one X chromosome in females. Initially, X inactivation was thought to be a random event. However, different degrees of escape combined with predominant inactivation of one X chromosome might explain the increased susceptibility of females to AIDs. A case–control study from the United States found a skewed inactivation of the X chromosome (arbitrarily defined as > 80%) in patients with GD and HT [118]. Although it is still not clear how the skewed inactivation influences the risk of GD, it is assumed that reduced expression of X chromosome-dependent self-antigens in the thymus primes inadequate T cell apoptosis [119]. Further, several PID genes are X-linked such as the genes for Wiskott–Aldrich syndrome protein, CD40L, or the IL-2 receptor gamma chain.

Differential histone methylation patterns may also be involved in peripheral tolerance. A report demonstrated specific patterns of hyper- and hypomethylation of histones in genes involved in T cell signaling (i.e., *CD247*, *LCK*, *ZAP70*, *CD3D*, *CD3E*, *CD3G*, *CTLA4*, and *CD8A*) in CD8^+^ and CD4^+^ T cells purified from whole blood [119]. Given the many signaling pathways involved, this results in vast possibilities with regard to potential influences on immune regulation.

The most potent form of epigenetic change is DNA methylation by a group of DNA methyltransferases at the 5’ carbon on the pyrimidine ring of cytosine using S-adenosyl-methionine as a co-factor [114]. This methylation process is usually permanent and results in a more condensed chromatin with reduced gene transcription. Many gene promoters in AIDs are hypomethylated, consequently enhancing transcription (e.g., PRF1 in SLE) [120]. A recent Chinese case–control study of genome-wide methylation reported that untreated female GD patients had significantly decreased methylation of the *ICAM-1* gene in leukocytes from whole blood [121]; as a consequence, they had higher ICAM-1 mRNA expression levels than normal controls. The authors concluded that this overall increased expression of ICAM-1 on blood cells may support the inflammatory process. However, this association has only been found in females and the results will also need to be replicated in patients of non-Asian descent.

As most studies reviewed here are based on a case–control design, the possibility cannot be excluded that epigenetic changes have occurred spontaneously during the course of treatment, have even been introduced by therapy, or that other co-variables, such as smoking, also play a role. Additionally, hyperthyroidism itself may be inducing epigenetic changes. Nevertheless, we are not aware of any longitudinal reports to date that might shed new light on this topic.

## Discussion

### Summary of evidence

Although there has been extensive research on the etiology of GD in the last decades, it has not been possible to identify a single causative agent, likely reflecting the fine balance between anergy and autoimmunity challenging our immune system throughout life.

Genetic influences have been extensively studied. Disappointingly, effect sizes of genetic predictors remain rather weak, with ORs of approximately 2, albeit with HLA-loci being the strongest. In a recent systematic review, we summarized the different genetic susceptibility factors [122]. Environmental influences seem to account for approximately 20% of the disease susceptibility. A modification of risk factors before disease onset would require a public health interventions and is unlikely to be accomplished. On the other hand, specific interventions altering environmental influences after onset may be beneficial in ameliorating disease course. Influences of overlapping factors, such as antibodies and cytokines, are hard to quantify. Nonetheless, they might offer a valuable link between the genetic and environmental factors as they are the most amenable to modification.

A diverse and multi-faceted etiology is more likely than a single causative agent, reflecting GD as a heterogeneous syndrome harboring elements of both immunodeficiency and autoimmunity. Consequently, future therapeutic strategies must consider multiple approaches to tackle this condition.

### Therapeutic outlook

Since their advent in the 1940s, ATDs have served as the backbone of GD treatment, although drug withdrawal results in high relapse rates (ranging from 30% to 60%) [4, 123]. Since their inception, it has been debated whether these drugs themselves harbor immunosuppressive properties, a hypothesis that has been supported by in vivo studies reporting reduced ICAM-1, IL-2, and IL-6 serum levels [4, 124]. Additionally, there is evidence from in vitro studies describing increased apoptosis of intrathyroidal lymphocytes and increased numbers of Treg cells, along with decreased HLA class II expression and decreased numbers of Th cells and natural killer cells during ATD therapy [124].

However, some findings contradict this hypothesis. First, in vivo immune changes are superimposed by a simultaneous resolution of hyperthyroidism. Secondly, if a true immunosuppressive effect of ATDs were to be present, one would expect a dose-dependent increase in remission rates. However, a Cochrane meta-analysis found that neither longer treatments nor higher doses with a block-and-replace regimen provided any benefit, but were instead associated with a higher rate of side effects [125].

Various cytokines could serve as biomarkers for GD or may even provide a therapeutic angle, for instance, by damping their action by anti-cytokine antibodies (ACAs) [126]. In AIDs, ACAs seem to have both beneficial and detrimental effects. A prototypic disease in this regard is pulmonary alveolar proteinosis, in which autoantibodies are directed against granulocyte macrophage colony-stimulating factor [127], inhibiting the differentiation and function of alveolar macrophages and resulting in deposition of lipids and proteins within the alveoli, causing recurrent infections and respiratory insufficiency. There are studies showing an inverse correlation between disease severity and ACA levels, for instance, in rheumatoid arthritis or Guillain–Barré syndrome, but there is also evidence indicating an increased disease severity with higher levels of ACAs, for instance in SLE [126]. In this light, a phase II multi-center trial in rheumatoid arthritis resistant to anti-TNF-α antibody treatment showed promising results [128] – patients treated with a TNF-kinoid not only developed neutralizing antibodies, but also had a trend towards improved clinical symptoms.

Reports with rituximab treatment (anti-CD20 antibody) in GD with or without ophthalmopathy have not been able to match expectations with regards to reduction in hyperthyroidism relapses or improved ophthalmopathy superior to that seen with glucocorticoids [87, 129, 130], likely due to the fact that rituximab targets only immature B cells, whereas mature plasma cells producing TRAb remain unharmed.

The fact that epigenetic modifications contribute to disease susceptibility and that the regulating steps are controlled by enzymes offers a potential new therapeutic approach [27]. In oncological indications such drugs have already been approved for treatment [131]. Although epigenetic drugs lack cellular specificity and thus have an increased potential for toxicity, they offer the advantage of intervening at a very early stage of the disease process [113].

TRAb are regarded as an integral part of the final common pathway since their presence inevitably leads to GD [13, 87]. Therefore, it could be envisaged that clearing these antibodies from circulation would result in rapid amelioration of disease. A method to clear the TRAb from the peripheral circulation could be the use of monoclonal antibodies directed against TRAb themselves. However, to the best of our knowledge, there are no such reports in the medical literature, except for an isolated publication from 1984 describing the formation of antibodies by injecting TRAb sera from patients into rabbits [132]. Nevertheless, in similar attempts in GD patients suffering from orbitopathy, administration of intravenous immunoglobulins of human origin did not show superior results compared with intravenous glucocorticoid treatment [133, 134]. Treatment with small molecule ligands blocking transduction of TSH-R signals through the binding to transmembrane helices and preventing their outward movement is further options, as is the use of antibodies that imitate TBII activity and prevent binding of TSAb by occupying the TSH-R [135]. An advantage of the final two options is the possibility of a combined effect on both thyroidal and orbital disease.

## Conclusions

Emerging evidence suggests that AIDs and diseases of immunodeficiency are not two distinct phenomena, but are in fact two sides of the same coin. This relationship has been repeatedly established in monogenetic diseases as IPEX (FOXP3 mutations) and in autoimmune polyendocrine syndrome type 1 (AIRE mutations). Moreover, polygenetic AIDs such as CVID or IgA-deficiency also exhibit multiple autoimmune phenomena, resulting in SLE, rheumatoid arthritis, and enteropathy. Thus, we believe that patients with GD in fact have either a genetically determined or secondarily acquired immunodeficiency that results in autoimmunity once certain triggering events occur.

Whereas both morbidity and mortality are high in other AIDs, GD is clinically much less severe. This being the case, there has been no urgent need for advances in the therapeutic regimen. Nonetheless, we believe that the current treatment approaches have a lack of specificity and can have undesired consequences (e.g., persistent hypoparathyroidism and laryngeal palsy in surgery or worsening of Graves’ orbitopathy in radioactive iodine treatment). There is thus a need for concerted efforts that warrant further investments.
